# Influence of Vectors’ Risk-Spreading Strategies and Environmental Stochasticity on the Epidemiology and Evolution of Vector-Borne Diseases: The Example of Chagas’ Disease

**DOI:** 10.1371/journal.pone.0070830

**Published:** 2013-08-08

**Authors:** Perrine Pelosse, Christopher M. Kribs-Zaleta, Marine Ginoux, Jorge E. Rabinovich, Sébastien Gourbière, Frédéric Menu

**Affiliations:** 1 Laboratoire de Biométrie et Biologie Evolutive UMR 5558, Centre National de la Recherche Scientifique, Université Lyon 1, Villeurbanne, France; 2 Mathematics Department, University of Texas at Arlington, Arlington, Texas, United States of America; 3 Public Health England, London, United Kingdom; 4 Ecologie et Evolution des Interactions UMR 5244, Centre National de la Recherche Scientifique, Université de Perpignan Via Domitia, Perpignan, France; 5 Centre for the Study of Evolution, School of Life Sciences, University of Sussex, Brighton, United Kingdom; 6 Centro de Estudios Parasitológicos y de Vectores, Universidad Nacional de La Plata, La Plata, Provincia de Buenos Aires, Argentina; Albert Einstein College of Medicine, United States of America

## Abstract

Insects are known to display strategies that spread the risk of encountering unfavorable conditions, thereby decreasing the extinction probability of genetic lineages in unpredictable environments. To what extent these strategies influence the epidemiology and evolution of vector-borne diseases in stochastic environments is largely unknown. In triatomines, the vectors of the parasite *Trypanosoma cruzi*, the etiological agent of Chagas’ disease, juvenile development time varies between individuals and such variation most likely decreases the extinction risk of vector populations in stochastic environments. We developed a simplified multi-stage vector-borne SI epidemiological model to investigate how vector risk-spreading strategies and environmental stochasticity influence the prevalence and evolution of a parasite. This model is based on available knowledge on triatomine biodemography, but its conceptual outcomes apply, to a certain extent, to other vector-borne diseases. Model comparisons between deterministic and stochastic settings led to the conclusion that environmental stochasticity, vector risk-spreading strategies (in particular an increase in the length and variability of development time) and their interaction have drastic consequences on vector population dynamics, disease prevalence, and the relative short-term evolution of parasite virulence. Our work shows that stochastic environments and associated risk-spreading strategies can increase the prevalence of vector-borne diseases and favor the invasion of more virulent parasite strains on relatively short evolutionary timescales. This study raises new questions and challenges in a context of increasingly unpredictable environmental variations as a result of global climate change and human interventions such as habitat destruction or vector control.

## Introduction

Environmental stochasticity is a major factor responsible for fluctuations in the density of populations, and possibly their extinction, and on an evolutionary timescale it strongly influences organisms’ life histories [1], [2]. Environmental stochasticity typically affects all individuals of a same cohort, developmental stage or population the same way, contrary to demographic stochasticity which may affect each individual differently [3]. Insects are particularly sensitive to variations in environmental factors such as temperature, which impacts their development rate [4], [5], rainfall, which determines the availability of larval habitats in mosquitoes [6], predation or habitat destruction. Unpredictable environmental variations are likely to increase as a result of global climate change [7] and other human interventions such as deforestations or pest and vector control. It is thus a fundamental question to what extent environmental stochasticity affects the distribution, epidemics, virulence and evolution of vector-borne diseases, i.e., diseases transmitted between hosts by blood-sucking arthropods, which are a major health threat in tropical and subtropical areas [8].

Several theoretical works have investigated the influence of different sources of random variability on the epidemiology and evolution of diseases. Demographic stochasticity in transmission rates and its effect on the epidemiology and evolution of directly-transmitted and vector-borne diseases have been well studied theoretically [9], [10], and efforts have also been made to account for demographic stochasticity in vector biodemographic rates in vector-borne diseases [11]. Climate-based transmission models that integrate explicit relationships between climatic factors and insect life-history traits such as development time have been developed to investigate the effect of climatic variations on the population dynamics of vectors and, in turn, on the epidemiology of vector-borne diseases [12]–[15]. However, to our knowledge, the possible impacts of environmental stochasticity on the evolution of vector-borne diseases have never been investigated.

Insects are well known to display specific behavioral and life-history strategies to face environmental uncertainty [16]–[18]. Life-history theory in stochastic environments predicts that strategies which spread the risk of encountering unfavorable conditions over time or space should be selected for in unpredictable environments [1], [2], [19], [20]. Density-dependent factors have also been shown to favor the evolution of risk-spreading (i.e., diversified bet-hedging) strategies [21], because they amplify population size variation and, in turn, the effects of environmental stochasticity. Iteroparity, which consists in reproducing multiple times over an organism’s lifetime, and inter-individual variability of juvenile development times due to dormancy variation are usual risk-spreading strategies reducing the probability of lineage extinction [22]–[24]. Such risk-spreading strategies exhibited by vector insects in stochastic environments could potentially affect the epidemiology, evolution and control of insect-borne diseases. This has been largely overlooked, except in a recent study suggesting that several species of triatomines, the vectors of *Trypanosoma cruzi*, the etiological agent of Chagas’ disease, exhibit, even under the same environmental conditions, an inter-individual variability of juvenile development times, and that such variability could have been selected as a response to environmental stochasticity [25]. The consequences of the variability in tick diapause duration on the epidemiology of tick-borne diseases have also been questioned [14]. However, the influence of such vector strategies on the prevalence and evolution of pathogen virulence has not been studied yet.

The epidemiology and evolution of parasites are affected by their hosts’ physiology, life-history traits, population biology and environment. In the case of vector-borne diseases, the availability of both hosts and vectors is likely to affect disease epidemiology and evolution [26]–[28]. Understanding how the interaction of these multiple factors affects parasite evolution, in particular their virulence, could permit the prediction of their impact on both humans and animals and help set up effective control strategies [29]. Theoretical and empirical works suggest that pathogens should evolve toward intermediate levels of virulence to their hosts, on the grounds that increasing replication rate within the host is beneficial in terms of increasing transmission probability, but also costly as it increases the level of damage to the host and therefore shortens the infectious period [29]–[38]. This compromise is referred to as the trade-off hypothesis between virulence and transmission [33], [34].

To what extent and how classical vector life-history traits such as developmental time, fecundity or survival could affect the evolution of parasite virulence to hosts is still not completely clear. Vector life-history traits do not seem to affect the long-term evolution of parasite virulence to hosts in a deterministic setting [39]. However, under stochastic settings, more complex host and vector population dynamics and extinctions might create ecological conditions favoring different parasite virulence strategies. In the case of a directly-transmitted disease submitted to demographic stochasticity, large host populations have been suggested to favor more virulent parasites as it seems less costly to harm one’s hosts when available hosts are numerous [40]. Environmental stochasticity associated to vector risk-spreading strategies could lead to similar results among vector-borne diseases.

Our work uses a theoretical modeling framework to test whether environmental stochasticity in interaction with vector life-history traits, in particular risk-spreading strategies, influences the epidemiology and evolution of vector-borne diseases. Our objective is to provide a general framework including only key demographic and transmission processes which could potentially apply to several vector-borne diseases [41]. We nevertheless have based our model on the specific example of triatomine biodemography, in particular the variability they display in the duration of their juvenile stage [25], [42]–[45]. Chagas’ disease is a chronic infection caused by the parasite *Trypanosoma cruzi* and transmitted to humans and other vertebrate hosts by blood-sucking triatomine insects [46]. This disease is a complex zoonosis covering a huge geographical area, submitted to diverse ecological and climatic conditions, and involving numerous host and vector species. Whereas it has long been restricted to rural Latin America areas where it is responsible for thousands of deaths each year, it is now a worldwide health threat because of increasing migratory flows, blood donations, organ transplants, and potential displacements of triatomines as a result of human activities and global climatic changes [47]–[49].

**Figure 1:**
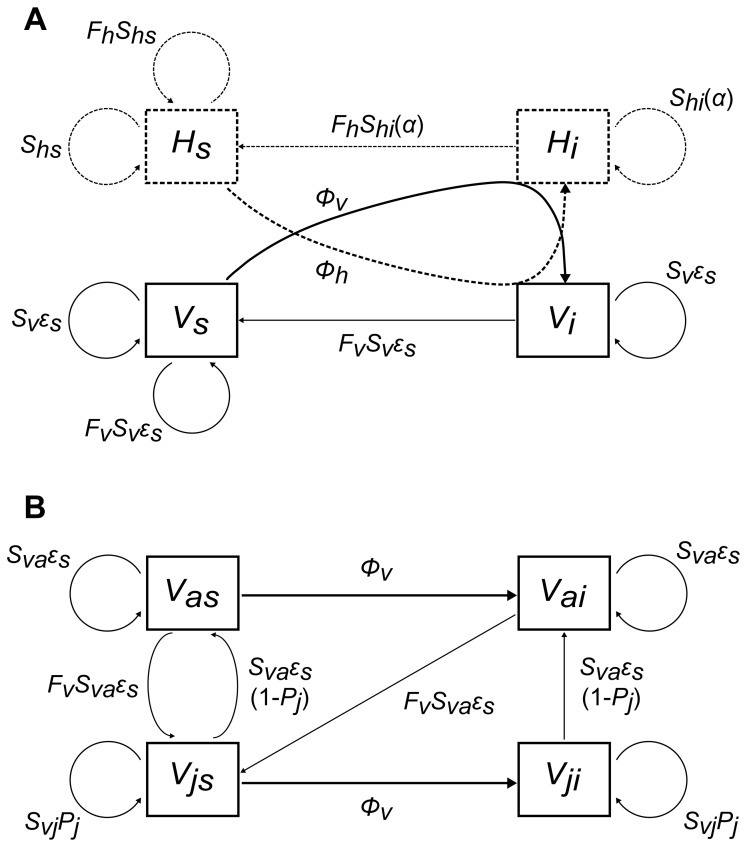
Schematic representation of the one-parasite-strain version of the model. Because vectors are divided into two stages (juvenile and adult), and that both stages can get and transmit the parasite, we present first a simplified vector-borne epidemiological model with only one stage for vectors (panel a) and then the vector life-cycle (panel b). Hosts are represented with dashed lines and vectors with solid lines (a) Susceptible hosts *H_s_* get infected through contacts with infected vectors *V_i_* with probability *Φ_h_*, and susceptible vectors *V_s_* through contacts with infected hosts *H_i_* with probability *Φ_v_*. Susceptible and infected vectors and hosts give birth to susceptible vectors and hosts. Infected host survival *S_hi_* is a function of parasite virulence *α*. Environmental stochasticity is applied to vector survival (only adults, see below) with intensity *ε_s_*. (b) Susceptible and infected adult vectors *V_as_* and *V_ai_* give birth to susceptible juvenile vectors *V_js_*. Susceptible and infected juvenile vectors *V_js_* and *V_ji_* remain in the juvenile stage with probability *P_j_* and mature into adults with probability (1-*P_j_*). Only adult survival *S_va_* is submitted to stochasticity with intensity *ε_s_*. See text, Table 1, Appendix S1 in File S1 and Fig. S1 for further details.

We developed a SI (i.e., Susceptible-Infected, with no recovery) vector-borne epidemiological model which considers for simplicity a single type of vectors and hosts, both regulated by density-dependent processes. Vectors in our model are structured in two explicit stages, a juvenile and an adult stage, both able to transmit the infection (Fig. 1, Fig. S1). Following previous work on triatomines’ developmental delay, we considered that vectors could display variable development times and that the juvenile stage was more resistant to environmental stress than the adult stage [25]. To create conditions under which displaying variable development times could lower vector population extinction probability, only adult vectors were subjected to environmental stochasticity. This generated risk-spreading strategies in vectors and we tested their influence on parasite epidemiology and evolution. Numerical simulations were carried out to compare the predictions of the model in a deterministic and a stochastic setting, in order to infer the role of environment stochasticity, as well as to be able to compare our results with other results obtained with deterministic models.

## Results

### The Influence of Vector Life-history Traits and Environmental Stochasticity on Vector Density and Parasite Prevalence

**Figure 2:**
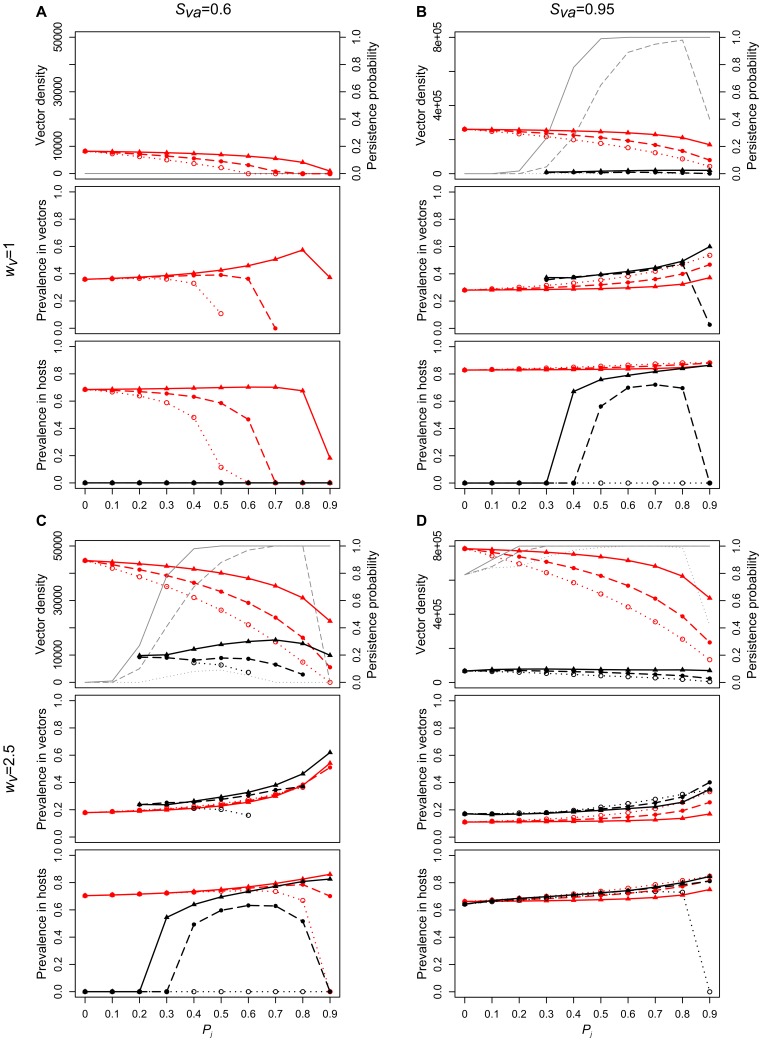
Influence of vector life-history traits on vector population dynamics and parasite prevalence. In each panel, the upper, middle and lower graphs display, respectively: total vector density, parasite prevalence in vectors, and parasite prevalence in hosts, according to the proportion of juvenile vectors prolonging the juvenile stage *P_j_*. Red and black lines correspond, respectively, to simulation results in the deterministic (shown at *t* = 150,000 weeks) and stochastic (shown at *t* = 10,000 weeks as median values over the 100 simulations, plotted only if the number of simulations without extinction is ≥5) case; dotted lines (open circles), dashed lines (closed circles) and solid lines (triangles) to a relatively low (*S_vj_* = 0.6), intermediate (*S_vj_* = 0.8) and high (*S_vj_* = 0.95) juvenile survival. Left and right panels correspond, respectively, to a relatively low (*S_va_* = 0.6; panels a, c) and relatively high (*S_va_* = 0.95; panels b, d) adult survival; upper and lower panels to a relatively low (*w_v_* = 1; panels a, b) and relatively high (*w_v_* = 2.5; panels c, d) fecundity. The persistence probability of vector populations (proportion of simulations for which vector density does not collapse before the end of the simulation), is given in grey in the upper graphs showing vector density. For prevalence among hosts, all simulations (including those for which vector populations collapse) are taken into account. For vectors (density and prevalence), only simulations for which vector population persisted until the end of the simulation are considered. Other parameters values are: *α_r_* = 0.008, *β* = 0.005, *c* = 0.01, *S_hs_* = 0.994, *w_h_* = 0.05, *g* = 100, *q* = 50, *b_max_* = 1, *ρ* = 0.5, *p_b_* = 0.2, *ε_S_* = 0.1.

As expected, vector life-history traits affect vector population density and, in turn, influence prevalence among vectors and hosts. Sensitivity analyses suggested that such effects were very similar whatever the level of parasite virulence to hosts *α* (results not shown). The virulence of the parasite introduced in the susceptible host - vector population was therefore set to *α_r_* = 0.008 in the entire epidemiological analysis. This value was selected as it corresponds to the typical level of virulence toward which the system converges after a long evolutionary time (see section “*Virulence evolution*” below). Among all possible combinations of vector life-history traits, we chose a sample of scenarios (Fig. 2) which accurately captures the diversity of the results observed in the whole study.

#### How do vector life-history traits affect vector density?

In the deterministic case, increasing the proportion *P_j_* of juvenile vectors prolonging the juvenile stage *P_j_* decreased of total vector density (Fig. 2, red lines). This result also held for stochastic environments, provided that the intensity of the stochasticity was relatively low (in this case, epidemiological patterns were, in general, very similar to the deterministic case, results not shown), or that vector life-history traits such as adult survival and fecundity were high enough to allow a high persistence of vector populations (Fig. 2d, black lines). Fewer juveniles molting into adults most likely lessens adult vector density and therefore the production of juveniles, which decreases total vector density. A decrease in other vector life-history traits (i.e., juvenile and adult survival and adult fecundity) led to a similar decrease in total vector density.

#### How does vector density affect parasite prevalence?

When vectors were relatively abundant, i.e., mostly in deterministic environments, and in stochastic environments when adult survival and fecundity were high (and as a consequence vector population persistence always high irrespective of *P_j_*), any decrease in vector density according to *P_j_* resulted in an increase of parasite prevalence in vectors but no (or limited) increase of prevalence in hosts (Fig. 2a, red lines, *S_vj_* high, *P_j_* low; Figs. 2b and 2d, red lines; Fig. 2c, red lines, *S_vj_* intermediate to high; Fig. 2d, black lines, *S_vj_* high, or *S_vj_* low but only for *P_j_* low). This unusual result can be explained by the fact that our model incorporates density-dependent mechanisms regulating vector population density. As vectors rely on blood meals and therefore compete for host access, decreasing vector density lessens the intensity of competitive interactions between vectors. This enhances the per-vector biting rate and seems to increase parasite prevalence among vectors. Conversely, at relatively low vector densities, decreasing vector density can reduce parasite prevalence among vectors and hosts (Fig. 2a, red lines, except for *S_vj_* = 0.95). Parasite prevalence among hosts obviously collapses when vector density approaches zero, even if parasite prevalence among the few remaining vectors could be relatively high (Fig. 2c, red lines, when *P_j_* higher than 0.6 and *S_vj_* lower than 0.95; Fig. 2d, black lines, *S_vj_* = 0.6 and *P_j_* = 0.9).

In stochastic cases, where *P_j_* drastically affects vector population persistence probability, the relationships between vector density and prevalence among vectors and hosts according to *P_j_* are more complex as no monotone patterns can be observed (Figs. 2b and 2c, black lines). These patterns are described in detail in the next section but we can already notice that great changes in vector or host prevalence can occur without major changes in vector density according to *P_j_*.

#### Risk-spreading strategies enhance parasite prevalence in stochastic environments

Risk-spreading strategies are defined as life histories maximizing the probability of persistence in unpredictable environments. Such risk-spreading strategies were observed in stochastic environments for combinations of vector life-history trait values depicted in black lines, in Figs. 2b and 2c. Under these conditions, we observed classical patterns of insect populations’ persistence according to risk spreading strategy [e.g. 1,2,24] (Figs. 2b and 2c, grey lines in graphs showing vector densities). This confirms that variable development times (i.e., *P_j_*≠0 and *P_j_*≠1) decrease the extinction risk when environmental stochasticity affects vector adult survival. Vector risk-spreading arose clearly for intermediate to high *P_j_* values, when either *S_va_* was relatively high and *w_v_* relatively low (Fig. 2b, dashed and solid grey lines) or *S_va_* relatively low and *w_v_* relatively high (Fig. 2c, dashed and solid grey lines). Under these conditions, low *P_j_* values always resulted in vector population extinction. For intermediate *S_vj_*, very high *P_j_* values also led to vector population extinction, as vector density became very low and eventually null (Figs. 2b and 2c, dashed grey lines). When *S_va_*, *S_vj_* and *w_v_* were too low, vector populations did not persist at any *P_j_* values (Fig. 2a, grey lines: vector persistence probability always null whatever *S_vj_*, and Figs. 2b and 2c, grey dotted lines: vector persistence probability always null when *S_vj_* = 0.6).

Vector risk-spreading strategies observed for intermediate to high *P_j_* values resulted in an increase in parasite transmission. Indeed, the prevalence according to *P_j_* followed the same patterns as vector population persistence probability: prevalence among hosts is maximal for intermediate or high values of *P_j_* (Figs. 2b and 2c, dashed and solid black lines). Note that when vector risk-spreading occurs, patterns of prevalence among hosts are very different than those among vectors. As a consequence, the latter cannot be used as a predictor of parasite prevalence among hosts.

#### Environmental stochasticity can enhance parasite prevalence as compared to deterministic contexts

Parasite prevalence among vectors was generally always slightly higher in the stochastic compared to the deterministic case (provided that vector persistence probability was not too low which resulted in very low to null prevalence). This can be explained by the fact that environmental stochasticity decreases vector density, and that when vector density is too high, this decrease results in higher parasite transmission. This result held for parasite prevalence among hosts in a few cases only. When vectors displayed no risk-spreading strategy, prevalence among hosts was always null or very low in the stochastic case, and therefore substantially inferior to the deterministic case (Figs. 2a, 2b and 2c). When vectors adopted risk-spreading strategies, prevalence among hosts remained lower in the stochastic case at intermediate *P_j_* values (Figs. 2b and 2c, dashed and solid lines), but reached values similar to the deterministic case when both *P_j_* and *S_vj_* were high (Figs. 2b and 2c, solid lines). When both *S_va_* and *w_v_* were high, prevalence among hosts was the same whatever the kind of environment (deterministic or stochastic) at low *P_j_* values, and at higher *P_j_* values was then slightly superior in the stochastic than in the deterministic case (Fig. 2d). In the same context, but with less stochasticity, prevalence among hosts was always slightly superior in the stochastic compared to the deterministic case independently of *P_j_* (results not shown).

### The Influence of Vector Risk-spreading Strategies on the Evolution of Parasite Virulence

Neither the proportion of juvenile vectors prolonging the juvenile stage *P_j_*, nor the other vector life-history traits and their interactions markedly influenced the value of the Continuously Stable Strategy (CSS) toward which parasite virulence converged over long evolutionary times. Furthermore, the CSS reached under the stochastic and deterministic settings were nearly the same. Nevertheless, the invasion speed of the mutant was affected by the interaction between the environment (i.e., stochastic or deterministic) and *P_j_*, which suggests that risk-spreading strategies in stochastic environments can affect the evolution of parasite virulence on shorter evolutionary timescales.

#### Effect of vector life-history traits and environmental stochasticity on the long-term evolution of parasite virulence

Simulations of the competition of resident and mutant parasites were carried out for all the demographic and epidemiological scenarios presented in the result section “*Epidemiology*”. Three types of environments were studied: deterministic, stochastic with relatively low stochasticity (*ε_S_* = 0.3) and stochastic with relatively high stochasticity (*ε_S_* = 0.1). In each environment, we considered all combinations of *P_j_* and other vector life-history trait values leading to a vector population persistence probability strictly superior to 5% (see Fig. 2), and a large range of *α_r_*/*α_m_* virulence couples (see the “*Model*” section). In all the tested cases, virulence always converged to an asymptotic CSS value of approximately *α* = 0.008 (result illustrated for a specific example in Appendix S2 in File S1 and Fig. S2). Therefore, vector life-history traits and vector risk-spreading strategies in stochastic environments do not influence the long-term evolution of parasite virulence. The virulence value obtained (*α* = 0.008) corresponds to an average life expectancy of 1 year and 5 months for infected hosts as compared to healthy hosts, which live an average of 3 years.

However, the time needed to reach mutant fixation appeared to vary between the cases studied (see Appendix S2 in File S1 and Fig. S2). As a consequence, we analyzed in more detail how the transient dynamics of mutant invasion was affected by vector risk-spreading strategies (see below).

#### Vector risk-spreading strategies affect the transient dynamics of mutant invasion

We analyzed the transient dynamics of mutant invasion for two cases for which vector risk-spreading strategies were previously identified: one corresponding to a relatively efficient *P_j_* (i.e., enhancing very significantly vector population persistence probability in stochastic environments) and the other one corresponding to a less efficient *P_j_* (see Figs. 2b and 2c, dashed and solid grey lines), in the stochastic versus deterministic case. We set mutant virulence *α_m_* to the CSS strategy of 0.008, so that the mutant is always expected to invade the resident in the long term, and we slightly varied *α_r_* around the mutant CSS strategy. We observed that environmental stochasticity and the level of efficiency of risk-spreading strategies can affect the transient dynamics of mutant invasion.

**Figure 3:**
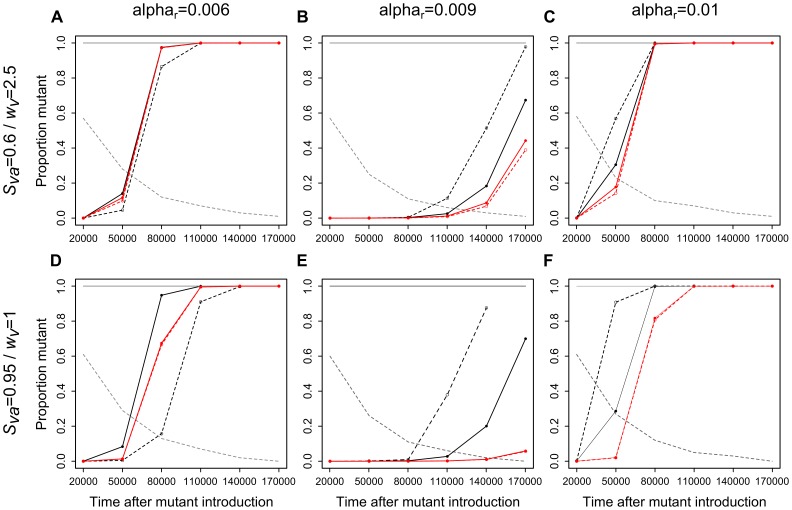
Dynamics of mutant invasion (proportion of mutants among infections according to time after mutant introduction). Proportion of the mutant is calculated as the number of hosts infected by the mutant parasite divided by the total number of infected hosts, and given as a median among all simulations for which the vector density did not collapse at the simulation time considered. Red and black lines correspond, respectively, to results in the deterministic and stochastic case (grey lines: persistence probability of vector populations); solid lines (solid circles) and dashed lines (open circles) to a relatively efficient and less efficient risk-spreading strategy. Panels a, b, c: *S_va_* = 0.6, *w_v_* = 2.5, less efficient risk-spreading strategy: *P_j_* = 0.3, more efficient: *P_j_* = 0.8; panels d, e, f: *S_va_* = 0.95, *w_v_* = 1, less efficient risk-spreading strategy: *P_j_* = 0.4, more efficient: *P_j_* = 0.8. Other parameters values are: *β* = 0.005, *c* = 0.01, *S_hs_* = 0.994, *w_h_* = 0.05, *g* = 100, *q* = 50, *b_max_* = 1, *ρ* = 0.5, *p_b_* = 0.2, *ε_S_* = 0.1, *S_vj_* = 0.95, *α_m_* = 0.008.

When *α_r_*>*α_m_*, the proportion of infections by the mutant parasite among all infected hosts during the transient phase of mutant invasion, or in other words, mutant invasion speed, was always higher in the stochastic (black lines) compared to the deterministic (red lines) case whatever the efficiency of vector risk-spreading strategy (Figs. 3b, 3c, 3e and 3f). Under stochastic environments, mutant invasion speed was also higher when vectors played a less efficient risk-spreading strategy (dashed black lines) as compared to a more efficient one (solid black lines, Figs. 3b, 3c, 3e and 3f).

Conversely, when *α_r_*<*α_m_*, mutant invasion was always slower in stochastic contexts in which vectors played a less efficient risk-spreading strategy (dashed black lines) compared to other contexts (Figs. 3a and 3d). Then, the invasion of the mutant in stochastic contexts in which vectors played a more efficient risk-spreading strategy (solid black lines) was either faster (Fig. 3d) or similar (Fig. 3a) than in deterministic environments (red lines). The difference in the results between Fig. 3a and Fig. 3d can be explained as follows: in the *S_va_* = 0.6/*w_v_* = 2.5 context (i.e., Fig. 3a), vector density at the time of mutant introduction when vectors adopted the efficient risk-spreading strategy (i.e., *P_j_* = 0.8) is approximately one half in the stochastic as compared to the deterministic environment (see epidemiological results on Fig. 2c), while in the *S_va_* = 0.95/*w_v_* = 1 context (i.e., Fig. 3d), it is approximately one tenth, which most likely boosted mutant invasion speed.

These results show that a mutant parasite less virulent than the resident invades faster under ecological contexts leading to a relatively high extinction probability of vector populations (i.e., in stochastic compared to deterministic environments, and with a less efficient compared to more efficient risk-spreading strategy). Conversely, a mutant parasite more virulent than the resident invades faster under ecological contexts leading to a relatively good persistence of vector populations (i.e., in deterministic environments and in stochastic environments with efficient risk-spreading strategies). This suggests that over relatively short evolutionary times, efficient risk-spreading strategies can favor more virulent parasites by enhancing vector population persistence probability.

## Discussion

Insects are sensitive to variations in environmental factors such as temperature, rainfall, predation, vector control, habitat change (e.g., pollution) or destruction. Climate variability and the frequency of extreme climatic events are most likely increasing with global climate change (e.g. [6], [7]). We therefore expect that insect risk-spreading strategies, that is, strategies able to reduce extinction risk in stochastic environments, will become more widespread in the future. Our work investigates theoretically, for the first time, the influence of environmental stochasticity and vector risk-spreading strategies, on both the epidemiology and evolution of vector-borne diseases. We showed that vector risk-spreading strategy in stochastic environments, defined here as an increase in the inter-individual variability in vector development time, can enhance parasite prevalence among both vectors and hosts (these changes being influenced by other vector life-history traits), but does not affect the long-term evolution of parasite virulence. On shorter timescales, they nevertheless affect the invasion speed of the mutant, less virulent parasites invading faster when the extinction risk of vector populations is high (i.e., in stochastic environments when vectors do not adopt efficient risk-spreading strategies), and more virulent parasites invading faster when vector population extinction risk is low (i.e., in deterministic environments, or when vectors adopt efficient risk-spreading strategies in stochastic environments). Our work therefore shows that environmental stochasticity and vector risk-spreading strategies are major factors that must be considered to understand both the epidemiology and short-term evolution of vector-borne diseases. It also strengthens the idea that vector biodemography in general has drastic consequences on the epidemiology of vector-borne diseases [41].

### Vector Risk-spreading Strategies in Stochastic Environments can Enhance Parasite Prevalence

Risk-spreading strategies are common and well described among insects (e.g. [22]–[24]) and seem to be displayed by triatomines, the vectors of the parasite *Trypanosoma cruzi* responsible for Chagas’ disease [25]. Our study shows that intermediate to high values of the vector development time parameter *P_j_* increase vector population persistence in stochastic environments, as they spread the risk that vectors reach the adult stage when a bad environmental event occurs. We showed that such risk-spreading strategies increase parasite prevalence among hosts compared to non risk-spreading strategies. More generally in our study, prevalence was either smaller or higher in stochastic compared to deterministic environments, depending on the values of vector life-history traits. Therefore, neglecting vector risk-spreading life histories may result in underestimating the epidemiological risk in stochastic environments, and deterministic models in general could lead to both over- and under-estimations of this risk.

Efficient risk-spreading strategies could be achieved in our model because only adult vectors were subjected to environmental stochasticity. Several lines of evidence suggest that triatomines’ juvenile stage, in particular the 5^th^ instar, could be more resistant than the adult stage to environmental stress such as starvation or insecticide exposure [see 25]. Smaller juveniles could also easily hide in small cracks in walls or in the ground and thus better resist stochastic events such as insecticide spraying, physical removal of vectors from human habitats, or predation. Such risk-spreading strategies and their epidemiological consequences may not be specific to triatomine vectors and Chagas’ disease. Indeed, variability in diapause duration, described in ticks and having a strong effect on tick population dynamics [14] could also possibly influence tick-borne disease transmission. Overall, our results suggest that vector temporal dispersion strategies and environmental stochasticity may play a role in the global increase of vector-borne disease epidemics and reemergence, such as the ones described in malaria [6].

### Risk-spreading Strategies Increase the Invasion Speed of more Virulent Parasites

Over a long evolutionary time, the vector life-history traits considered in our study do not influence the evolution of parasite virulence to hosts in either the deterministic or the stochastic settings. In this regard, our study is consistent with a previous modeling study conducted on dengue in a deterministic setting which showed that vector life-history traits do not influence the long-term evolution of virulence to hosts [39]. Our work extends this result to the stochastic context, includes juvenile variable development time, and considers a density-frequency-dependent evolutionary process, which is different from this existing study that has used a “*R_0_* optimization approach” (see [29]). In vector-borne diseases, in the same manner as in directly-transmitted disease systems, the only life-history trait which seems to influence the long-term evolution of virulence to hosts is host background mortality, long-lived hosts giving rise to long-lasting infections and selecting for less virulent pathogens, and short-lived hosts selecting for more virulent pathogens [39], [71]–[73]. We reached the same conclusion with our model: an increase in host survival decreased the value of the evolutionary stable virulence (results not shown). Further work, which falls beyond the scope of the present study, should look in more detail at the determinants of *T. cruzi* virulence, in particular under the influence of host biodemography.

The study of the initial stages of the competition between pathogen strains is very relevant in the context of emerging diseases (e.g. [40]), but also when extinctions driven by stochastic processes preclude looking at the asymptotic strategies reached after long evolutionary times, as is traditionally done in deterministic models. Our analysis of the transient dynamics of the invasion of a mutant parasite reinforces this idea. Indeed, we showed that vector risk-spreading strategies in stochastic environments differentially affect, on relatively short timescales, the invasion speed of parasite strains depending on their virulence level. In particular, when vector population extinction risk is high, the invasion speed of less virulent parasites (i.e., less harmful to the hosts) is increased. More important, our model also predicts that efficient vector risk-spreading strategies create favorable conditions for the rapid invasion of more virulent pathogen strains, as they improve vector persistence probability. A similar conclusion could be reached with a directly-transmitted disease model including demographic stochasticity: dense host populations that provide less restrictive conditions for pathogen invasion selected for increased virulence [40].

Pathogen extinction rates in vector-borne systems, for which vectors are more severely subjected to environmental stochasticity than hosts, is probably lower than in directly-transmitted disease systems, because hosts might provide reservoirs for the pathogen [74]. Lastly, vector metapopulation dynamics, in particular their immigration and/or recolonization of treated areas can have drastic consequences on vector persistence [75]–[79] and pathogen transmission [40], [80], [81], and might also be used as a strategy to decrease vector extinction risk. Similarly, vector immigration might select for more virulent pathogens, as it decreases the local risk of extinction [82].

### Decreasing Vector Abundance Usually Increases Parasite Transmission, via a Decrease in Vectors’ Competition for Host Access

The inclusion in our model of a density-dependent mechanism for regulating vector populations revealed novel aspects of the effects of vector demography on parasite prevalence. Most previous epidemiological models assume that transmission rate is an increasing function of host density. Vector-borne models traditionally assume that the vector per host ratio affects transmission rates, but that hosts are numerous and therefore do not constitute a limiting resource for vectors which can feed at their preferred rate (e.g. [26], [27]). However, in Chagas’ disease, as probably in other vector-borne diseases, vectors are known to compete for host access, and this most likely regulates vector populations [53]–[56]. The vector per host ratio and processes of saturation in contacts between hosts and vectors have a strong influence on the transmission dynamics [28], [60], [61] and the evolution of the parasite (at least in the specific case of the evolution of transmission modes, see [83]).

Here we showed that as a result of vector competition for host access, decreasing vector density can increase parasite prevalence in vectors and hosts (unless vector density is too low). Consequently, because environmental stochasticity results in a decrease in vector density, prevalence among vectors is often higher in the stochastic version of our model compared to the deterministic one, and there are a few cases for which prevalence among hosts is also slightly higher. This contrasts with previous models including demographic stochasticity that usually lead to lower prevalence compared to deterministic models (e.g. [9]). Few comparisons between the predictions of stochastic versus deterministic epidemiological models have been carried out, especially among models with an evolutionary component (but see [10]), and our work is original in this respect.

Our model assumes that vector biting rate increases when vector density relative to hosts decreases and that the quantity of blood ingested is constant. As an alternative, we could have considered that when vectors are too numerous relative to hosts, host defensive behaviors interrupt blood meals and therefore reduce their size. This would reduce the infectivity of the bites because parasite transmission requires that feces are deposited in the vicinity of the bite. This alternative should therefore lead to similar predictions because infection probability would increase when the competition between vectors decreases.

### Conclusion

Our work is the first to investigate the epidemiological and evolutionary consequences of the interaction between vector risk-spreading strategies and environmental stochasticity. Our results show they must be taken into account in epidemiological and evolutionary studies on vector-borne diseases and strengthen the idea that vector-borne diseases are strongly affected by vector biodemography [41]. Our model bears properties specific to the Chagas’ disease system, the main one being that both juvenile and adult stages are able to transmit the parasite. This applies also to tick-borne diseases as all ticks’ developmental stages are infective. We believe that a global approach, taking into account adaptive biodemographic responses of vectors to environmental stochasticity when trying to understand the epidemiology and evolution of vector-borne diseases, should be considered and applied more widely to other systems.

## Methods

We built a discrete-time SI model describing triatomines’ life-cycle (juvenile and adult stage) and vector-borne transmission of *T. cruzi* to a single host species. To investigate the epidemiological dynamics of the model, and in particular the influence of vector life-history traits on disease prevalence in hosts and vectors, a one-parasite-strain model was used. This model has a total of 6 classes (Fig. 1). To investigate the evolutionary dynamics of parasite virulence, we used a two-parasite-strain version of the model considering that a resident *r* and a mutant *m* compete for host access (no co-infection was considered). This model has a total of 9 classes. The equations of the two-parasite-strain model are given in Appendix S1 in File S1. We first describe the deterministic form of the model, and then explain how environmental stochasticity was applied.

We used a time step of 1 week as triatomines feed once about every five-ten days under optimal, laboratory conditions (see [50] and below). At each time step, a certain proportion of hosts and vectors first survive, then produce newborns (host and vector newborn mortality is incorporated in the fecundity), and then eventually become infected by one of the two parasite strains (see schematic sequence of events of the one-parasite-strain version of the model in Fig. S1). Vectors are divided into a juvenile and an adult stage and, at each time step, a proportion *P_j_* of juvenile vectors remain in the juvenile stage. Both triatomine larvae and adults are haematophagous, and consequently both are able to contract and transmit the infection. For simplicity, we assume that there is no difference in the two stages’ infectivity and susceptibility to infection. Hosts and vectors produce susceptible offspring even if infected. Even if vertical transmission has been reported in a few host species (e.g. in mice [51]; in humans [52]), we consider its role in the transmission of Chagas’ disease to be negligible. For simplicity, we also assume that hosts and vectors turn infective immediately after becoming infected and that their infectivity remains unchanged whatever the time since infection.

### Host Biodemography

*T. cruzi* infects numerous vertebrate hosts (sylvatic and domestic) that show contrasted life-history trait values (e.g. life expectancy, fecundity). Taking into account multiple host populations was technically difficult and our work focuses on the biodemography of vectors. We therefore considered a single “hypothetical” host for which *T. cruzi* is pathogenic, *i.e.* the parasite generates a mortality cost. The proportion of susceptible hosts surviving at each time step is assumed to be *S_hs_* = 0.994 (which corresponds, in terms of probabilities, to an approximate life expectancy of 1/0.006 = 167 weeks or 3.1 years) (see Table 1 for all parameter values used in the model). The survival probability of infected hosts is the probability that they survive from “natural causes”, *S_hs_*, and the probability that they survive from the infection. We define virulence *α* as the probability to die as a result of the infection. By extension, and because in our model, virulence is assumed to vary according to the parasite strain (resident or mutant), the proportion of infected hosts surviving at each time step is considered to be:
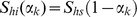
with *k* referring to the resident or mutant strain. In our model, and as is commonly assumed in models of pathogen virulence, virulence is therefore defined as an additive mortality cost.

**Table 1:** Definition and values of the parameters used in the model.

Parameter	Definition	Value (or range of values) used in the model
*S_hs_*	Proportion of hosts surviving at each time step.	0.994
*ω_h_*	Maximal number of offspring per host per time step.	0.05
*g*	Density dependence factor for hosts: number of hosts at which host fecundityis reduced by two.	100
*S_va_*	Proportion of adult vectors surviving at each time step (parameter submittedto stochastic variations).	variable (from 0.6 to 0.95)
*S_vj_*	Proportion of juvenile vectors surviving at each time step.	variable (from 0.6 to 0.95)
*P_j_*	Proportion of juvenile vectors prolonging the juvenile stage at each time step.	variable (from 0 to 0.9)
*ω_v_*	Maximal number of eggs per vector per time step.	variable (1; 2.5) [58], [59]
*q*	Density dependence factor for vectors: V/H ratio at which vector fecundityand biting rate is divided by two.	50
*b_max_*	Maximal (preferred) biting rate of vectors (in bites per vector per time step).	1 [62]
*α_k_*	Probability of the host to die as a result of an infection by the parasite strain *k* (virulence).	0.008 in the epidemiological approach; variable according to parasite strain in the evolutionary approach
*β_h_*	Stercorarian* transmission probability (host infection probability) (in infected hosts per bite).	0.005
*c*	Shape of the function linking virulence and transmission rate.	0.01
*ε_S_*(*t*)	Quantity by which vector adult survival is multiplied (*ε_S_* = 1 during “good” periods and *0≤ εs ≤1* during “bad” periods), or intensity of stochasticity.	variable (0.1; 0.3)
*p_B_*	Probability that a bad period occurs.	0.2
*ρ*	Autocorrelation coefficient among good and bad periods.	0.5

* transmission of the parasite to the host via the feces of the infected vector.

Susceptible and infected hosts are assumed to display the same density-dependent fecundity, *F_h_*(*t*):
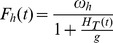
where *ω_h_* is the maximum number of offspring produced per host and per time step, *g* the density of hosts at which host fecundity is divided by two, and *H_T_*(*t*) the total host density, i.e., the sum of susceptible and infected (whatever the parasite strain) host density. We assume that each female host produces a maximum of 5 newborns per year (approximately 0.1 per week). Both males and females are considered in our model, and assuming a 0.5 sex-ratio, *ω_h_* = 0.05.

### Vector Biodemography

Contrary to hosts, vectors do not usually suffer a mortality cost when infected by *T. cruzi* (see [46] for a review). The proportion of adult and juvenile vectors surviving at each time step are *S_va_* and *S_vj_*, respectively, and the proportion of juvenile vectors prolonging the juvenile stage is *P_j_* (see Table 1 for the range of parameter values used). A value for *P_j_* strictly between 0 and 1 creates a temporal variability in the time at which juveniles reach the adult stage. Increasing *P_j_* increases both the average and variability of the juvenile stage duration. To survive (and for adult vectors, to reproduce as well), vectors need to ingest blood from hosts (these host-vector contacts may result in parasite transmission when one of two partners is susceptible and the other one infected, see below). Hosts cannot tolerate an unlimited amount of bites: they are known to become irritable and defend themselves against vectors when they receive too many bites [53]–[56]. Increasing the vector per host ratio *Q*(*t*) = *V_T_*(*t*)/*H_T_*(*t*) (*V_T_*(*t*) being the total vector density including all infection status and developmental stages) could therefore result either in blood meals being interrupted by host defensive behaviors, which would lessen the average quantity of blood ingested, or in a global reduction in the average vector biting rate if access to hosts is made difficult. Because triatomines cannot produce eggs without ingesting blood meals but can undergo long starving periods, we assume, following [57], that the vector per host ratio *Q*(*t*) affects vector fecundity but not survival. Triatomines’ fecundity is therefore assumed to be a decreasing function of *Q*(*t*) and is expressed as:
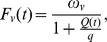
with *ω_v_* being the maximum number of eggs produced per vector and per week and *q* the *Q* ratio for which vector fecundity is divided by two. Under laboratory conditions, fecundity estimates from triatomines of the genus *Triatoma* vary between 2 and 6 female eggs per female per week (e.g. [58], [59]). To take into account the sex-ratio and egg mortality, we set *ω_v_* to either 1 or 2.5 in our analysis (see Table 1 for the parameter values used in the model).

### Infection Dynamics

Vector-borne models, and in particular models of Chagas’ disease transmission (e.g. [27]) usually follow the assumption developed by Ross [26] on malaria that hosts are always numerous and vectors feed at their preferred rate. As reported above, hosts can however constitute a limited resource when the number of vectors per host is large. We assumed for simplicity that the quantity of blood ingested was unaffected by the vector per host ratio and that the per-vector biting rate (in number of bites per vector per time step) saturates for low *Q* values and decreases gradually as *Q* increases [28], [60], [61], which leads to the following expression:
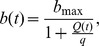
with *b_max_* being the maximum (preferred) vector biting rate (in number of bites per vector per time step). Data on triatomine spontaneous drive for food in the field are lacking and are likely to be highly variable among triatomine species. In the laboratory, when triatomines are offered a host daily, the preferred feeding frequency averages one blood meal every 5–10 days for species of the triatomine genus *Rhodnius* [62]. For simplicity, and because in the laboratory feeding conditions are optimal for the insects, we set *b_max_* = 1, i.e., vectors feed at a maximum rate of one time per week. New infections in hosts require a contact between a susceptible host and an infected vector. The number of new hosts infected by a parasite strain *k* at each time step is thus calculated as the product of the total number of blood meals per time step, *b*(*Q*(*t*))*V_T_*(*t*), the proportion of contacts involving a susceptible host *H_s_*(*t*)/*H_T_*(*t*), the proportion of contacts involving a vector (either adult or juvenile) infected by the strain *k*, *V_ik_*(*t*)/*V_T_*(*t*), and the probability *β_h_* that such a contact gives rise to an infection in the host (in infected hosts per bite) [28]:



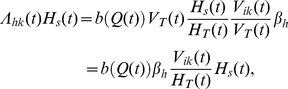
Λ*_hk_*(*t*) being the rate of new host infections by the parasite strain *k*. The total probability for a host to become infected by one of the two strains (resident *r* and mutant *m*) is therefore:



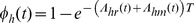
with *T* being time in weekly units. To keep this total bounded between 0 and 1, and to avoid coinfections, we define the probability for a host to become infected by strain *k* as:



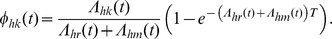



In a similar way, new infections in vectors require contacts between susceptible vectors and infected hosts. If we set *β_v_* as the probability that such a contact gives rise to an infection in the vector (in infected vectors per bite), the number of new vectors infected by a parasite strain *k* per time step is:
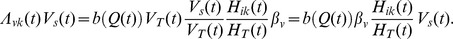



This leads to the following expression for the probability for a vector to become infected by strain *k*:
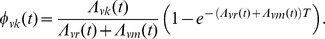



Resident and mutant parasites are assumed to differ in the intensity of the harm they induce to their host, *i.e.* virulence *α* (defined in our model as an additive mortality cost to natural mortality). It is well accepted in the literature that virulence is correlated to the growth rate of the parasite inside the host, *i.e.* to the total number of pathogen particles present in the host. Increasing virulence therefore typically leads to an increase of pathogen transmission probability from infected hosts to vectors *β_v_*, up to a certain threshold [30]–[32]. In our model, we therefore expressed *β_v_* as a simple increasing function of *α* with decreasing benefits, as has been traditionally done in the literature (e.g. [29], [33]):
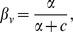

*c* being the shape parameter of this function.

### Environmental Stochasticity

Our main objective is to study the combined influence of vector risk-spreading strategies and environmental stochasticity on the epidemiology and evolution of the disease. Because risk-spreading strategies most likely occur at the juvenile stage in triatomines [25], and in order to create conditions under which variability in the juvenile stage duration decreases extinction risk under unpredictable environmental conditions, only adult vectors are subjected to environmental stochasticity in our model. We chose to expose adult vector survival rather than fecundity to stochasticity. Indeed, as triatomines are iteroparous (i.e. they have several reproductive periods during their lifetime), subjecting their reproduction to stochasticity has only a limited impact on vector population densities.

In the stochastic version of our model, a stochastic sequence of “good” and “bad” periods was considered, “bad” periods occurring with probability *p_B_*. Because the time step of one week chosen here is relatively small, and switching from a “good” to a “bad” period every week might be unrealistic, we define a parameter *ρ*, between 0 and 1, controlling the intensity of the autocorrelation between periods. The probability to switch from a “good” to a “bad” period is set to *p_B_.(1-ρ)* and from a “bad” to a “good” period to *(1-p_B_).(1-ρ)* [63]. The greater *ρ* is, the more difficult it is to switch from one type of period to another. At each time step of the simulation process, a number was sampled from a uniform distribution (0,1), and switching occurred when it was strictly less than the corresponding switching probability (as given above). *ρ* and *p_B_* have been set to the values 0.5 and 0.2, respectively, as calibrations showed that the sequence of “good” and “bad” periods under these parameter values corresponds roughly to seasonal variations. At each time step, the proportion *S_va_* of adult vectors surviving is multiplied by *ε_S_*(*t*), which is set to 1 during “good” periods and to a value 0≤*ε_S_*<1 during “bad” periods. *ε_S_* therefore controls the intensity of the environmental stochasticity (with *ε_S_* = 0 leading to the death of adults when a “bad” period occurs, see Table 1 for the parameter values used in the model).

### Analysis of the Influence of Vector Life-history Traits on Parasite Epidemiology

To assess how vector life-history traits influence infection dynamics (and in a second step the parasite’s evolutionary dynamics, see below), numerical simulations were performed in both deterministic and stochastic settings. For the epidemiological analysis, a single individual infected by a resident parasite was introduced in a susceptible host – vector population. Juvenile vectors were always chosen as the class in which parasites arise (both resident and mutant), as simulations showed that the system converged toward the same prevalence and evolutionary strategy regardless of the class in which the mutation was introduced (results not shown). Susceptible host, adult and juvenile vector initial densities (i.e., numbers per unit area) have been set to 100, 100 and 200, respectively, for all simulations. The spatial unit used is on the order of 1 km^2^. The output variables of the epidemiological analysis are the prevalence of the parasite (among vectors and hosts) and the population densities at *t* = *t_m_* = 10,000 time-steps in the stochastic and *t* = *t_m_* = 150,000 time-steps in the deterministic case (*t_m_* being the time at which the mutant is introduced, see below). Preliminary work not shown here determined that these time frames were long enough in the stochastic case for the disease to spread in the population and short enough to avoid all vector populations collapsing (host populations never collapse as they are not exposed to stochasticity), and long enough for the equilibrium state to be reached in the deterministic case. For each parameter combination tested, 100 simulations were performed in the stochastic case. During all simulations in our study (for both epidemiological and evolutionary steps), we avoided unrealistic situations in which extremely small population densities (e.g. on the order of 10^−10^) persisted over very long times by replacing by 0 all densities falling below an arbitrary value of 10^−5^. Furthermore, at the end of the simulation times, we considered populations were extinct if their density was less than 10^−3^.

### Analysis of the Influence of Vector Life-history Traits on Parasite Evolution

To assess whether vector life-history traits influence the evolutionary dynamics of parasite virulence, we followed the Adaptive Dynamics framework [29], [64]–[66], and introduced a mutant at time *t_m_*, in the host – vector population already infected by the resident parasite. This second (evolutionary) step consists of a competitive interaction between a mutant and a resident parasite for infection of susceptible hosts and vectors. We assessed the evolutionary dynamics of parasite virulence strategy by analyzing the outcome of the competition for combinations of resident and mutant virulence strategies (*α_r_* and *α_m_* were varied from 0 to 0.016 with a 0.002 step, a range corresponding to the same average life expectancy to a life expectancy divided by almost 4, for infected hosts compared to healthy hosts). An Evolutionarily Stable Strategy (ESS) is defined as a strategy that cannot be invaded by any other strategy [67], and a convergent-stable strategy as a strategy attainable throughout the course of evolution via small mutational steps [68]. A strategy verifying both properties is a Continuously Stable Strategy (CSS, [69]; see [70] for further details). Compared to a “*R_0_* optimization approach” in which the evolutionary stable strategy is sought as the strategy which maximizes the basic reproduction number *R_0_*, or parasite fitness, this approach considers that the adaptive value of a strategy depends on the strategies played by other individuals (here, parasites), and the frequency of these strategies (see [29] for further details). Because during the first (epidemiological) step involving the resident only, some vector populations collapsed among the 100 initial simulations, we increased, when necessary, the number of initial simulations in order to have always 100 simulations at the time of introduction of the mutant *t_m_*. In our analysis of the long-term evolution of virulence (see section “*Results*”), the outcomes of the competition between mutant and resident parasite strains for all *α_r_*/*α_m_* virulence couples were captured at *t* = *t_m_*+20,000, *t* = *t_m_*+50,000, *t* = *t_m_*+80,000 and *t* = *t_m_*+170,000 weeks in the stochastic case (in order to find the best balance between stochastic extinctions and parasite strain fixations), and at *t* = *t_m_*+170,000 weeks in the deterministic case. In our analysis of the dynamics of mutant invasion on a relatively shorter term (see section “*Results*”), outcomes of the competition between mutant and resident parasites were captured, in both the deterministic and stochastic cases, at times varying from *t* = *t_m_*+20,000 to *t* = *t_m_*+170,000, using steps of 30,000 weeks.

## Supporting Information

Figure S1 **Sequence of events between time** ***t*** **and** ***t+1*** **in the one-parasite-strain model.**
(PDF)

Figure S2 **Pairwise Invasibility Plots.**
(PDF)

File S1 **Appendix S1,** Equations of the two-parasite-strain system. **Appendix S2,** Long-term evolution of parasite virulence.
(DOC)
