# Supplementary figures and images for: Influence of Vectors’ Risk-Spreading Strategies and Environmental Stochasticity on the Epidemiology and Evolution of Vector-Borne Diseases: The Example of Chagas’ Disease
Source: PLoS One. 2013 Aug 8;8(8):e70830. doi: 10.1371/journal.pone.0070830 (PMC3738595; doi:10.1371/journal.pone.0070830)

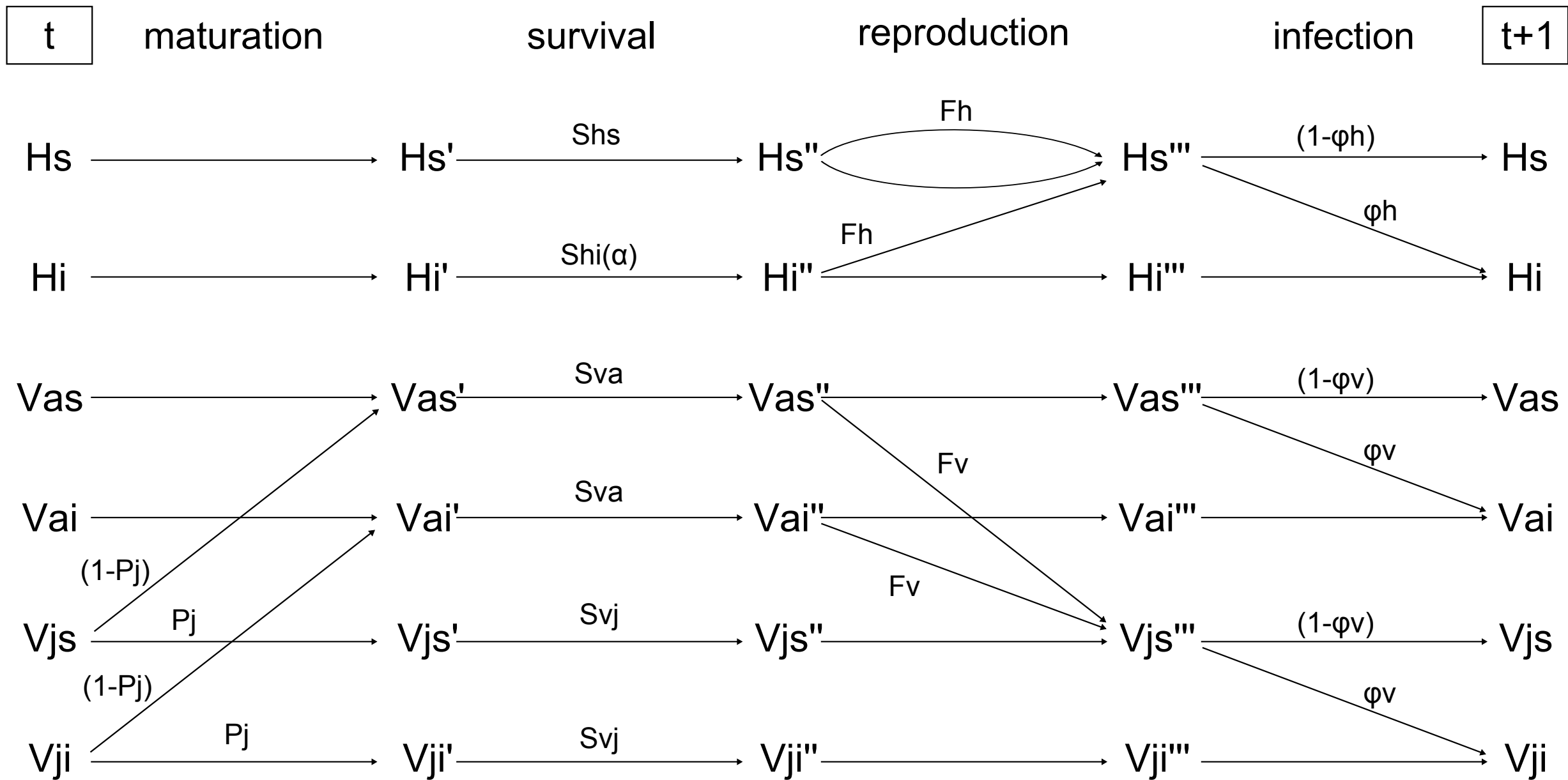

Supplement: Figure S1 — Sequence of events between time t and t+1 in the one-parasite-strain model. (PDF) [file pone.0070830.s001.pdf]

# Deterministic

$P_j=0.3$

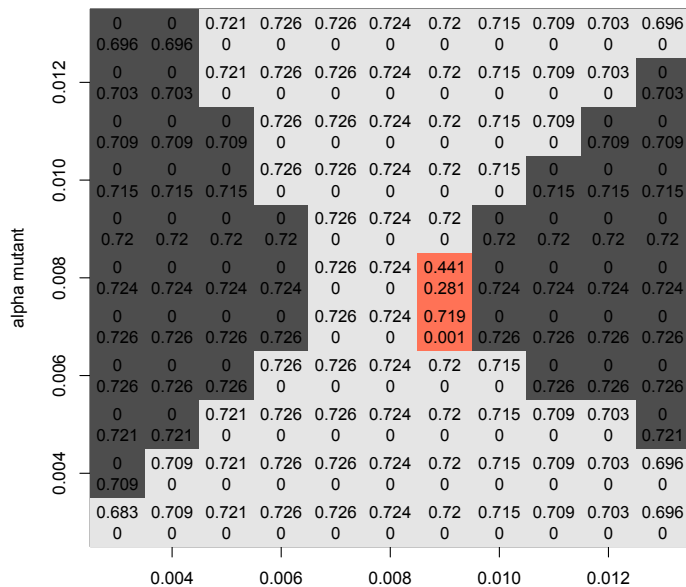

# Stochastic

alpha mutant

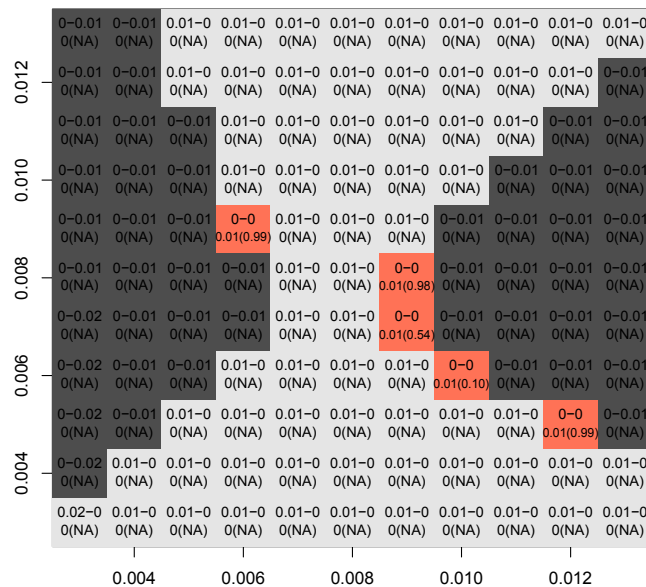

$P_j=0.8$

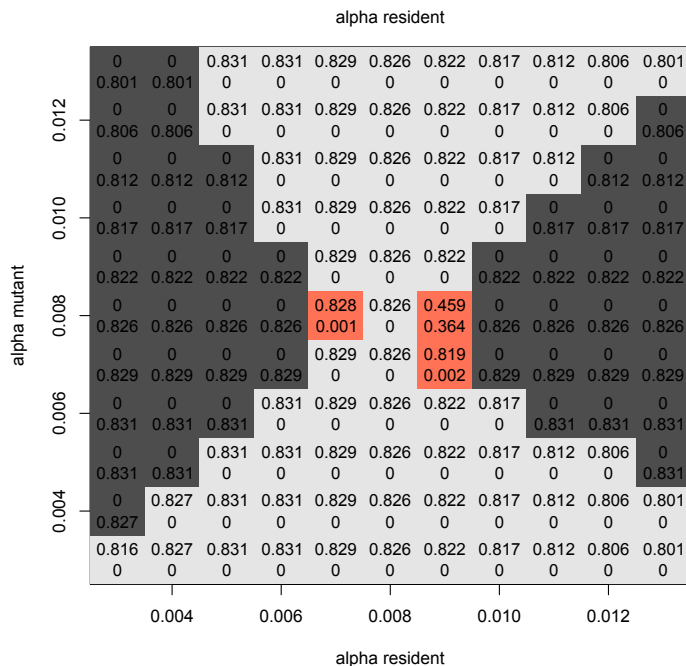

alpha mutant

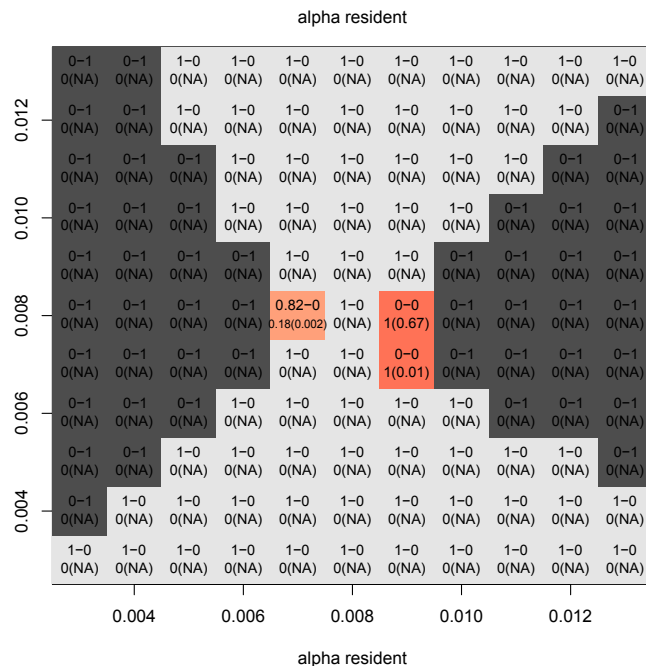

Supplement: Figure S2 — Pairwise Invasibility Plots. (PDF) [file pone.0070830.s002.pdf]
