# Supplementary material for: Influence of Vectors’ Risk-Spreading Strategies and Environmental Stochasticity on the Epidemiology and Evolution of Vector-Borne Diseases: The Example of Chagas’ Disease
Source: PLoS One. 2013 Aug 8;8(8):e70830. doi: 10.1371/journal.pone.0070830 (PMC3738595; doi:10.1371/journal.pone.0070830)
Supplement: File S1 — Appendix S1, Equations of the two-parasite-strain system. Appendix S2, Long-term evolution of parasite virulence. (DOC) [file pone.0070830.s003.doc]

**Influence of vectors’ risk-spreading strategies and environmental stochasticity on the epidemiology and evolution of vector-borne diseases: The example of Chagas’ disease**

Perrine Pelosse^1,2,3,*^, Christopher M. Kribs-Zaleta^2^, Marine Ginoux^1^, Jorge E. Rabinovich^6^, Sébastien Gourbière^4,5^, Frédéric Menu^1^.

^1^ Laboratoire de Biométrie et Biologie Evolutive UMR 5558, Centre National de la Recherche Scientifique, Université Lyon 1, Villeurbanne, France.

^2^ Mathematics Department, University of Texas at Arlington, Arlington, Texas, USA.

^3^ Public Health England, London, UK.

^4^ Ecologie et Evolution des Interactions UMR 5244, Centre National de la Recherche Scientifique, Université de Perpignan Via Domitia, Perpignan, France.

^5^ Centre for the Study of Evolution, School of Life Sciences, University of Sussex, Brighton, UK.

^6^ Centro de Estudios Parasitológicos y de Vectores, Universidad Nacional de La Plata, La Plata, Provincia de Buenos Aires, Argentina.

^*^ E-mail: perrine.pelosse@gmail.com

**Supporting Information**

**Appendix S1**

The equations of the two-parasite-strain system are:

where the following variables are expressed as densities (numbers per unit area) at time *t* (parameters definitions are given in the main body of the article): *H_s_*(*t*): susceptible hosts, *H_ir_*(*t*): hosts infected by resident, *H_im_*(*t*): hosts infected by mutant, *V_as_*(*t*): susceptible adult vectors, *V_air_*(*t*): adult vectors infected by resident, *V_aim_*(*t*): adult vectors infected by mutant, *V_js_*(*t*): susceptible juvenile vectors, *V_jir_*(*t*): juvenile vectors infected by resident, *V_jim_*(*t*): juvenile vectors infected by mutant.

**Appendix S2**

We illustrate that the interaction of vector life-history traits and environmental stochasticity does not influence the long-term evolution of parasite virulence with a specific combination of parameters that depicts well what was observed throughout the entire study (*ε_S_*=0.1, *S_va_*=0.6, *w_v_*=2.5, *S_vj_*=0.95, Fig. 2c, solid lines, in the main body of the article). Under this demographic-epidemiological context, we selected the two risk-spreading *P_j_* values 0.3 and 0.8, as the first one is less effective and the second one more effective in enhancing vector population persistence probability under stochastic environments. For *P_j_*=0.3, the vector persistence probability is approximately 0.8 and the prevalence in hosts approximately 0.55, and for *P_j_*=0.8, the vector persistence probability is 1 and the prevalence in hosts is approximately 0.8 (cf. Fig. 2c, in the main body of the article).

Under this demographic-epidemiological context, as under the other contexts tested throughout the study, *P_j_* did not markedly affect the Continuously Stable Strategy (CSS) virulence value, and no significant difference was observed between the deterministic and stochastic cases. The pairwise invisibility plots (PIPs) in all cases displayed the same global configuration, with virulence strategies around 0.008 that cannot be invaded by any other strategies and that can be reached by small evolutionary steps (i.e. gradual small mutations) (see Supplementary Fig. S2).

The resolution of the PIPs around 0.008 depends on the step chosen for *α*, because in our numerical simulations we varied *α* discretely with a relatively small 0.001 step that corresponds to approximately 1 month. One can notice that on the main diagonal (*α_r_*=*α_m_*) of the PIPs, the resident parasite always outcompete the mutant parasite. Under classical Adaptive Dynamics, neutral mutations, however, lead to an equal number of residents and mutants, as both strategies are equally advantageous. Under our modeling conditions, the first strategy to appear therefore seems to benefit from a priority effect [1].

Even if the vector strategy and environmental stochasticity did not influence the global shape of the PIPs, fine differences could nevertheless be observed around the CSS value. In particular, in the stochastic case, *P_j_* and whether *α_m_* is inferior or superior to *α_r_* seemed to affect the speed at which mutant invasion took place, as the interaction of these two factors affected mutant proportion at time *t*=*t_m_*+170,000. This motivated our analysis of the transient dynamics of mutant invasion (see the main body of the article). In particular, on the left side of the CSS (i.e., *α_r_*<*α_m_*), mutant invasion seemed to be faster in the *P_j_*=0.8 than in the *P_j_*=0.3 case, and on the right side of the CSS (i.e., *α_r_*>*α_m_*), faster in the *P_j_*=0.3 than in the *P_j_*=0.8 case. For instance, for the *α_r_*=0.007 / *α_m_*=0.008 couple, the mutant parasite was still absent in the *P_j_*=0.3 case (light grey cell) and present in 18% of the simulations coexisting with the resident parasite in the *P_j_*=0.8 case (light orange cell, median prevalence mutant=0.002). For the *α_r_*=0.009 / *α_m_*=0.008 couple, the mutant parasite was always present coexisting with the resident parasite in both cases (dark orange cells), but with a median prevalence of 0.98 in the *P_j_*=0.3 case and 0.67 in the *P_j_*=0.8 case (vector population persistence is only 1% at *t_m_*+170,000 when *P_j_*=0.3, while it is 100% when *P_j_*=0.8, but this results holds for shorter evolutionary times and therefore higher persistence proportions for *P_j_*=0.3, see the main body of the article).

**Supplementary Figure Legends**

**Figure S1. Sequence of events taking place between time *t* and *t+1* in the one-parasite-strain model.**

*H*, *V_a_* and *V_j_* are the densities (i.e., numbers per unit area) of hosts, adult vectors, and juvenile vectors, respectively. The subscripts *s* and *i* stand for susceptible and infected, respectively. *P_j_*: proportion of juvenile vectors prolonging the juvenile stage at each time step; *S_hs_*: proportion of susceptible hosts surviving at each time step; *S_hi_(α)*: proportion of infected hosts surviving at each time step (as a function of parasite’s virulence *α*); *S_va_*: proportion of adult vectors surviving at each time step; *S_vj_*: proportion of juvenile vectors surviving at each time step; *F_h_*: host fecundity; *F_v_*: vector (adult) fecundity; *φ_h_*: host probability of infection, when bitten by an infected vector; *φ_v_*: vector probability of infection, when feeding on infected host.

**Figure S2. Pairwise Invasibility Plots (PIPs).**

“Pictures” of the outcomes of the competition between mutant and resident parasites are shown in both the deterministic (left graphs) and stochastic (right graphs) settings, and for *P_j_*=0.3 (graphs on first row) and *P_j_*=0.8 (graphs on second row), at *t*=*t_m_*+170,000 (the mutant introduction time in the stochastic and deterministic cases are *t_m_*=10,000 and *t_m_*=150,000, respectively). X-axis: resident parasite’s virulence strategy *α_r_*; y-axis: mutant parasite’s virulence strategy *α_m_*. Cell colors give the competition outcome of an *α_r_* / *α_m_* couple: dark grey cells: mutant replaced resident (over the entire set of simulations for which the vector population did not collapse in the stochastic case); light grey cells: mutant did not replace resident and only resident present at the end of the simulation (over the entire set of simulations in the stochastic case); orange cells: both mutant and resident persisted at the end of the simulation. Under stochastic environments: dark orange cells: both mutant and resident persisted over the entire set of simulations; light orange cells: mixture of both mutant and resident present and only resident present at the end of simulations. Numbers inside the cells represent, in the deterministic case: resident (upper number) and mutant prevalence (lower number); in the stochastic case: proportion of simulations among initially run simulations for which resident only remained (upper left number), mutant only remained (upper right number), both mutant and resident remained (lower left number). In the stochastic case, among simulations in which both mutant and resident remained: the median proportion of the mutant, i.e., number of hosts infected by mutant among all infected hosts (lower right number in parentheses). Evolutionary dynamics of *α* can be assessed as follows. For the *α_r_*=0.005/*α_m_*=0.006 couple, a dark grey color indicates that the mutant wins the competition. It therefore becomes resident. If we proceed by small-step mutations, we reach the *α_r_*=0.006 / *α_m_*=0.007 couple, and a dark grey color indicates again that the mutant wins the competition. While on the left-hand sides of the PIPs, an increase in *α* is advantageous for the mutant parasite (i.e., allows the replacement of the resident parasite), on the right-hand sides, a decrease is advantageous. The four PIPs therefore predict that parasite strategy converges towards *α*=0.008, and that once the resident strategy is set to *α*=0.008, no other mutant can outcompete the resident. This strategy is both attainable by small-step mutations and non-invadable, it is thus a CSS strategy. Other parameters values are: *β*=0.005, *c*=0.01, *S_hs_*=0.994, *w_h_*=0.05, *g*=100, *q*=50, *b_max_*=1, *ρ*=0.5, *p_b_*=0.2, *ε_S_*=0.1, *S_va_*=0.6, *w_v_*=2.5, *S_vj_*=0.95.

**Reference**

1. Gourbière S, Menu F (2009) Adaptive dynamics of dormancy duration variability: evolutionary trade-off and priority effect lead to suboptimal adaptation. Evolution 63: 1879–1892.
